# A GCG expansion (GCG)_11_ in polyadenylate-binding protein nuclear 1 gene caused oculopharyngeal muscular dystrophy in a Chinese family

**Published:** 2011-05-25

**Authors:** Juan Ye, Huina Zhang, Yandan Zhou, Han Wu, Changjun Wang, Xin Shi

**Affiliations:** Department of Ophthalmology, the 2nd Affiliated Hospital of Zhejiang University, College of Medicine, Hangzhou, Zhejiang, China

## Abstract

**Purpose:**

To identify the mutation in polyadenylate-binding protein nuclear 1 gene (*PABPN1*, previously termed *PABP2*) in a Chinese family with autosomal, dominantly inherited oculopharyngeal muscular dystrophy (OPMD).

**Methods:**

Clinical and ophthalmologic examinations were conducted on available living family members from three generations. Genomic DNA was extracted from peripheral blood leukocytes of every available family member, and the fragment flanking the (GCG)_n_ of the *PABPN1* gene was amplified by PCR. Mutations were screened by DNA sequencing. Photographs of deceased family members were examined for signs of OPMD.

**Results:**

Clinical features of OPMD were found in all patients in generation II except the youngest sister, and no clinical manifestations were found in generation III. Mutation sequencing demonstrated that (GCG)_6_ in the wild *PABPN1* gene was expanded to heterozygous (GCG)_11_ in all affected family members and in some but not all unaffected members.

**Conclusions:**

In a Chinese family with autosomal dominantly inherited OPMD, a heterozygous (GCG)_11_ expansion was identified in all affected family members and in several young unaffected members.

## Introduction

Oculopharyngeal muscular dystrophy (OPMD) is an autosomal dominant late-onset disease that is characterized by progressive bilateral ptosis, dysphagia, and dysarthria [1]. It is a rare disease with a prevalence of approximately 1/100,000 worldwide [2]. In 1962, Victor et al. [3] first described a family with OPMD; nine members of three generations were known to be affected (OPMD; OMIM 164300**).** In a large cohort in New Mexico, ptosis was reported to be the first symptom to appear, sometimes concurrently with dysphagia. Pharyngeal weakness and gait abnormalities were common and occurred later than ocular weakness [4]. Although the disease is mainly inherited in an autosomal dominant manner, an autosomal recessive mode of transmission was suggested as early as 1975 [5].

In 1998, Brais et al. [6] identiﬁed a stable trinucleotide repeat expansion at the N-terminus of the *PABPN1* gene on chromosome 14q11 in affected families. The normal allele is (GCG)_6_ repeat-encoding a polyalanine (polyA) stretch at the 5′ end, but the dominant expansion can range from (GCG)_8_ to (GCG)_13_, and the autosomal recessive form of the disease occurs as a single GCG expansion, (GCG)_7_. Adjacent to the (GCG)_6_ repeat, there is a GCA GCA GCA GCG coding sequence. GCG and GCA both encode alanine molecules (Ala), so the wild PABPN1 protein has a stretch of 10 Ala, whereas the mutated PABPN1 in dominant OPMD has 12–17 Ala in the N-terminal domain.

To date, Hung-Chou Kuo et al. [7] have reported the (GCG)_6_(GCA)(GCG)_4_ mutation in a Taiwanese family, and a Chinese Malaysian case with the (GCG)_9_ mutation has been reported by Khean Jin Goh et al. [8]. Recently, You et al. [9] reported the identical mutation, (GCG)_9_(GCA)_3_GCG, in a mainland Chinese family with OPMD. Herein, we describe another mainland Chinese family with an autosomal dominant form of OPMD, in which we detected a heterozygous (GCG)_11_(GCA)_3_GCG expansion.

## Methods

### Patient and family data

**Figure 1:**
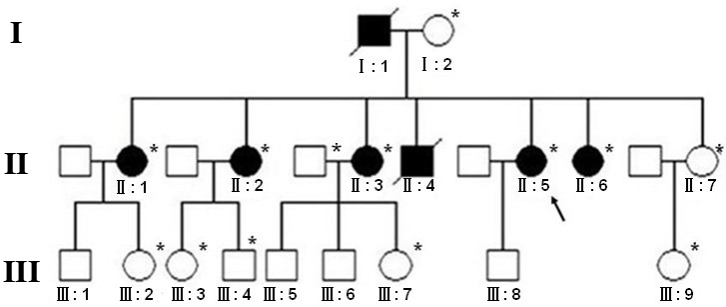
Pedigree of the family. Squares represent male. Circles represent females. Affected individuals are indicated by black circles and squares. Unaffected individuals are indicated by white circles and squares. The arrow points at II-5, the proband. Diagonal lines indicate the deceased. *The patient has been examined.

The proband (II-5) in this family presented with ptosis. She was 54 years old, and bilateral eyelid drooping had gradually developed since age 47. She did not notice the drooping until it affected her vision. She had to tilt her head backward and use the frontalis muscle to raise her eyelid, severely affecting cosmesis. Later, the patient also had dysphagia and dysarthria. The pedigree (Figure 1) was constructed based on interviews with family members and examination of photographs of deceased family members. All members of generation II were affected, except the youngest sister (II-7), who was 41 years old. None of the nine individuals in generation III (aged 12–41 years) was affected yet.

### Genomic DNA preparation

All procedures followed the tenets of the Helsinki declaration, approval was obtained from the Local Research Ethics Committee of Zhejiang University, and written informed consent was obtained from all patients in the study. Blood specimens (4 ml) from all of the patients and available family members (marked with an asterisk in Figure 1) were collected in a BD Vacutainer (Becton-Dickinson, Franklin Lakes, NJ) containing EDTA. Genomic DNA was extracted from leukocytes of peripheral blood using a QIAmp blood kit (Qiagen, Hilden, Germany).

### PCR and DNA sequencing

A pair of special primers (forward: 5′-GGC AGG CAG CTT GAC TAA TG-3′; reverse: 5′-CTC AGA CTC CAG GCC GTT CTA-3′) were designed to amplify the fragment flanking the GCN repeat. The cycling conditions were 98 °C for 30 s, followed by 35 cycles at 98 °C for 10 s, 57 °C for 5 s, 72 °C for 20 s, and finally 72 °C for 5 min and 20 °C for 5 min, using PrimeSTAR HS DNA polymerase and 2× PrimeSTAR GC Buffer (Mg^2+^ Plus; TaKaRa, Japan). The PCR product was isolated by electrophoresis on a 2% agarose gel and sequenced using the BigDye Terminator Cycle sequencing kit V 3.1 (ABI Applied Biosystems, Foster City, CA) on an ABI PRISM 3730 Sequence Analyzer, according to the manufacturer’s directions.

## Results

### Clinical analysis

**Figure 2:**
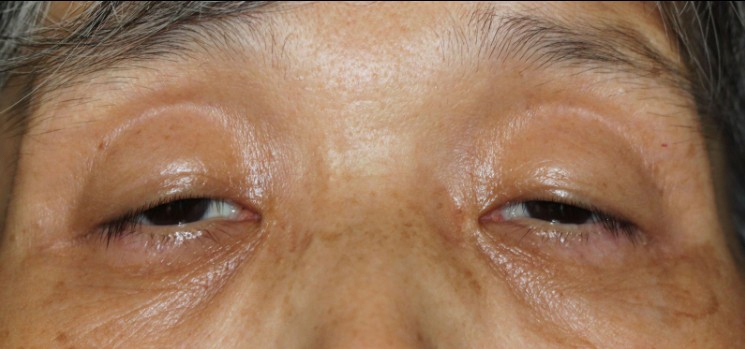
Photograph of II-5. She is 63 years old, has had gradual progressive ptosis since she was 50, and suffers a facial weakness.

Clinical and genetic information was obtained from 12 family members (11 women, 1 man; Table 1). All of the affected family members had begun to develop bilateral and symmetric eyelid drooping in approximately their fifth decade, followed later by dysphagia and dysarthria, and the affected members recalled that their father (I-1) also developed drooping eyelids when he was approximately 50 years old. The oldest sister (II-1) could barely see, since even when she used the frontalis muscle, the fissure of palpebrae was only 3 mm and the levator function was 1 mm (Figure 2). She had difficulty swallowing solid foods, such as certain vegetables. II-4 died in a traffic accident at age 40, and he had not developed severe ptosis by that time.

**Table 1:** Clinical data and GCG expansion of available family members.

**Patient number**	**Gender**	**Age (years)**	**Initial symptom**	**Age of onset (years)**			**(GCG) expansion**
**Ptosis**	**Dysphagia**	**Dysarthria**
I-2	F	87	-	-	-	-	N
II-1	F	63	Ptosis	50	51	52	11/6
II-2	F	61	Ptosis	48	50	50	11/6
II-3	F	60	Ptosis	50	51	51	11/6
II-5	F	54	Ptosis	47	50	50	11/6
II-6	F	52	Ptosis	45	48	50	11/6
II-7	F	41	-	-	-	-	N
III-2	F	33	-	-	-	-	11/6
III-3	F	32	-	-	-	-	11/6
III-4	M	30	-	-	-	-	N
III-7	F	22	-	-	-	-	N
III-9	F	15	-	-	-	-	N

Abbreviations: F: female; M: male; +: present; -: absence; N: normal.

Because of the symmetric nature of ophthalmoplegia, these patients did not complain of diplopia. They might have been unaware of their decreased motility until it became severe.

### Molecular genetic analysis

**Figure 3:**
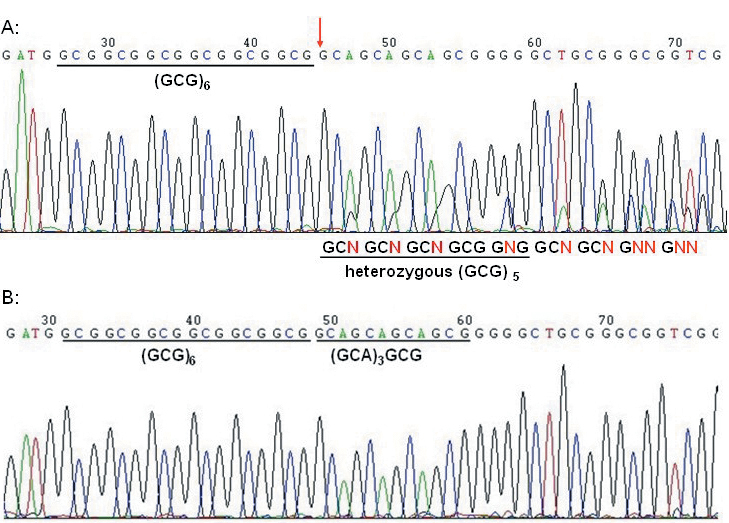
PCR product sequencing chromatograms. **A**: The patient’s heterozygous (GCG)_11_ expansion mutation is shown; the red arrow points to the start of the frameshift, and before it are the normal (GCG)_6_ sequences. The heterozygous alleles are bimodal and marked “N”. **B**: The wild-type *PABPN1* gene sequence from a healthy Chinese people is shown.

After sequencing the PCR product of the *PABPN1* gene, we identified a heterozygous (GCG)_11_ repeat in all the affected individuals of the II generation. The youngest sister (II-7, 41 years old), who was not yet affected, revealed a normal allele. In the III generation (ages 12–41 years), no family members were affected, but among the examined members, III-2 and III-3 showed the same heterozygous (GCG)_11_ repeat as the affected family members; other individuals showed a normal trinucleotide repeat (GCG)_6_. The mutation was not detected in 100 unrelated Chinese people without OPMD, who served as controls (Figure 3).

## Discussion

Based on the family history, clinical manifestations, and molecular genetic analysis, our family was easily diagnosed with OPMD inherited in an autosomal dominant manner. Genetic diagnosis is very important for patients suspected of having OPMD. Although creatine kinase and L-lactate dehydrogenase are reported to be generally moderately increased in most patients with OPMD, the patients in our study demonstrated normal levels (data not shown), as has been reported in two Japanese families heterozygous for the (GCG)_11_(GCA)_3_GCG allele with OPMD [10]. It is unknown why the elevation of serum creatine kinase is absent only in Asian (GCG)_11_ expansion mutation patients.

OPMD occurs worldwide, particularly in the French-Canadian population. Barbeau [11] showed that all of the numerous reported French-Canadian cases could be traced back to a single ancestor who emigrated from France in the 1600s. Currently, several types of (GCG)n expansion have been reported, ranging from (GCG)_8–13_, with (GCG)_9_ being seen most frequently. The (GCG)_11_ type had previously been reported by Nagashima et al. [10] and Uyama et al. [12] in three Japanese families.

Nagashima et al. described a big family with 54 members, ten of whom were affected through three generations. Bilateral ptosis developed in the fourth and fifth decades in the lives of the affected family members, who later developed diplopia, nasal voice, dysphagia, and muscle weakness. Uyama et al. [12] reported two unrelated families, including 30 individuals affected by OPMD through four generations. They developed bilateral ptosis, most of them in their fifth decade, followed by dysphagia and limb weakness. All three families had disease severity, for example, onset of ptosis, similar to ours. If haplotype, including the number of GCG repeats, is identical among those families, those families might have a common ancestor, and we are conducting further research. It should be noted that the (GCG)_7_ allele can act as a recessive mutation. *PABPN1* gene is the first example of a relatively frequent allele that can act either as a modifier of a dominant phenotype or as a recessive mutation [5].

Although most published reports have described the insertion as (GCG)n, some studies have identified GCA repeat within the expanded GCG sequence [13–15], and this is seen mainly in the English population. Besides the insertion mutations, a special point mutation was reported by Robinson et al. [16]. The mutation was a c.35G>C base change resulting in a glycine-to-alanine substitution at amino acid position 12 (p.Gly12Ala). Thus, the 10 alanine-glycine-2 alanine was in lieu of 13 contiguous alanines, which is the cause of disease in the common triplet repeat expansion mutation.

Trinucleotide expansions are common in human pathologies. They can be classified according to the amino acid they code for: polyglutamine (polyQ) repeat diseases and polyalanine (polyA) repeat diseases [17]. PolyQ diseases include Huntington’s disease, spinobulbar muscular dystrophy, spinocerebellar ataxias types 1,2,3 (or Machado-Joseph disease), and so forth [18]. The polyQ disease often contains a large group of trinucleotide repeats (>35), whereas OPMD is a unique disease that contains short trinucleotide expansions (>2) but shares the features of polyQ and other polyA diseases. Their common feature is protein misfolding and subsequent aggregation [19]. The mutation is thought to confer a toxic gain-of-function on the protein. Abu-Baker et al. [17] have recently proposed that the expansion of the polyA repeat in mutant PABPN1 protein causes misfolding and exposes the hydrophobic alanine stretch, which would otherwise be buried inside the protein in the wild-type form. Thus, the longer the polyA stretch is, the more exposed the hydrophobic region is. Hill et al. [20]reported that in the Caucasian population in South England, the onset age of the most dominant form of OPMD, of which expansions range from(GCG)_8_ to (GCG)_13,_ ranges from 40 to 70 years, with a mean of 51 years. However, in the recessive models, of which the expansion is (GCG)_7_, the age of onset is always between 69 and 74 years [2]. Repeat length appears to correlate with disease severity, suggesting a relationship between genotype and phenotype. The wild *PABPN1* gene encodes for 10 Ala residues in protein, whereas the mutant alleles in our patient encode 15 Ala instead of 10. Thus, the number of alanine residues in our patients are equivalent to those reported to be generated by the (GCG)_6_(GCA)_3_(GCG)_2_(GCA)_3_GCG mutation [21].

Another contrast between polyA and polyQ is the mutational mechanism. In polyQ diseases, polymerase slippage has long been assumed to be the mechanism. Polymerase slippage means the slipping of DNA polymerase III from the DNA template strand at the repeat region and its subsequent reattachment at a more distant site during replication. Polymerase slippage can cause the newly created DNA strand to contain an expanded section of DNA. However, in the mutation of OPMD, the expansions are small and stable through mitosis and meiosis, making unequal recombination more likely [22]; this is a rare event, accounting for the stability of mutations over many generations, as suggested by Nakamoto et al. [23].

In conclusion, we have identified a recurrent, short GCG expansion (GCG)_11_ in the *PABPN1* gene in an Asian family with a phenotype characterized by ptosis as the first symptom. This is the first report of a Chinese family with OPMD caused by the (GCG)_11_ mutation of the *PABPN1* gene. Molecular genetic analysis is a valuable method for the diagnosis of OPMD.
